# Supplementary material for: Broadly neutralizing SARS-CoV-2 antibodies through epitope-based selection from convalescent patients
Source: Nat Commun. 2023 Feb 8;14:687. doi: 10.1038/s41467-023-36295-5 (PMC9907207; doi:10.1038/s41467-023-36295-5)
Supplement: Supplementary file 1 — Supplementary Information [file 41467_2023_36295_MOESM1_ESM.pdf]

Broadly neutralizing SARS-CoV-2 antibodies through epitope-based selection from convalescent patients

Supplementary Information

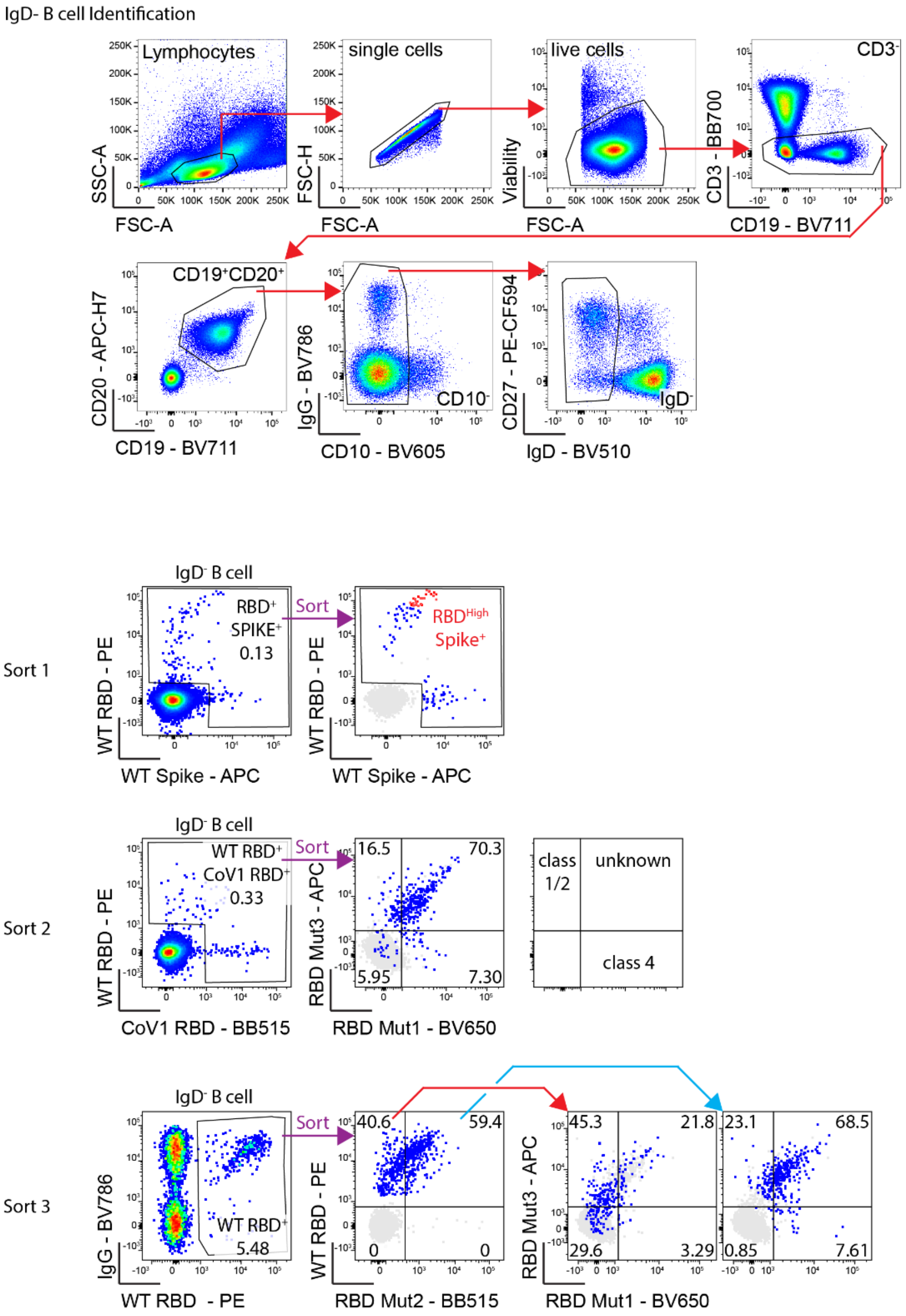

Supplementary Fig. 1: Overview of the PBMC sorting strategy. Representative flow cytometric plots of circulating IgD- CD27+ memory B cells in a convalescent patient one month after SARS-CoV-2 infection. The cells were also stained with fluorescent tetramers of SARS-CoV-2 RBD and SARS-CoV-2 spike protein for the Sort 1 strategy. For the Sort 2 and Sort 3 strategies, cells were strained with WT RBD, Mut1, Mut2 and SARS-CoV-1 RBD (replaced by Mut3 RBD in Sort 3).

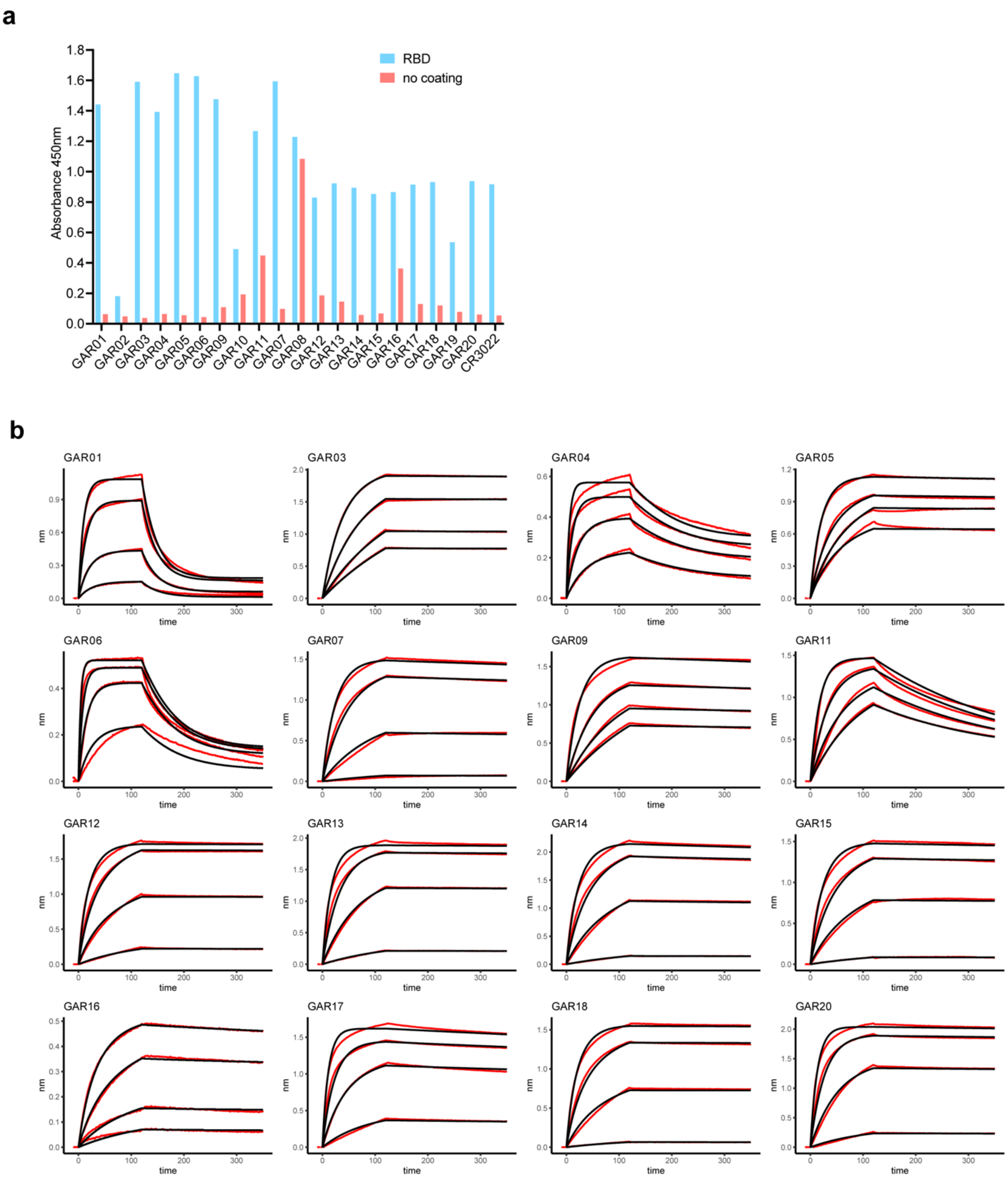

Supplementary Fig. 2: Binding of monoclonal antibodies to SARS-CoV-2 RBD. a: Purified antibodies were initially screened by ELISA for their binding to the RBD. RBD was coated on a Maxisorp ELISA plate at 2 µg/ml, incubated first with the purified antibody at 100 µg/ml and then with HRP conjugated to anti-human IgG. **b**: Affinity measurements by BLI. Streptavidin sensors were coated with biotinylated antibodies at 40 µg/ml, and association and dissociation constants measured using 2-fold serial dilutions of the SARS-CoV-2 RBD from 400 to 50 nM. Raw binding curves are represented in red and the fitted curves in black (K_D values in Fig. 1c).

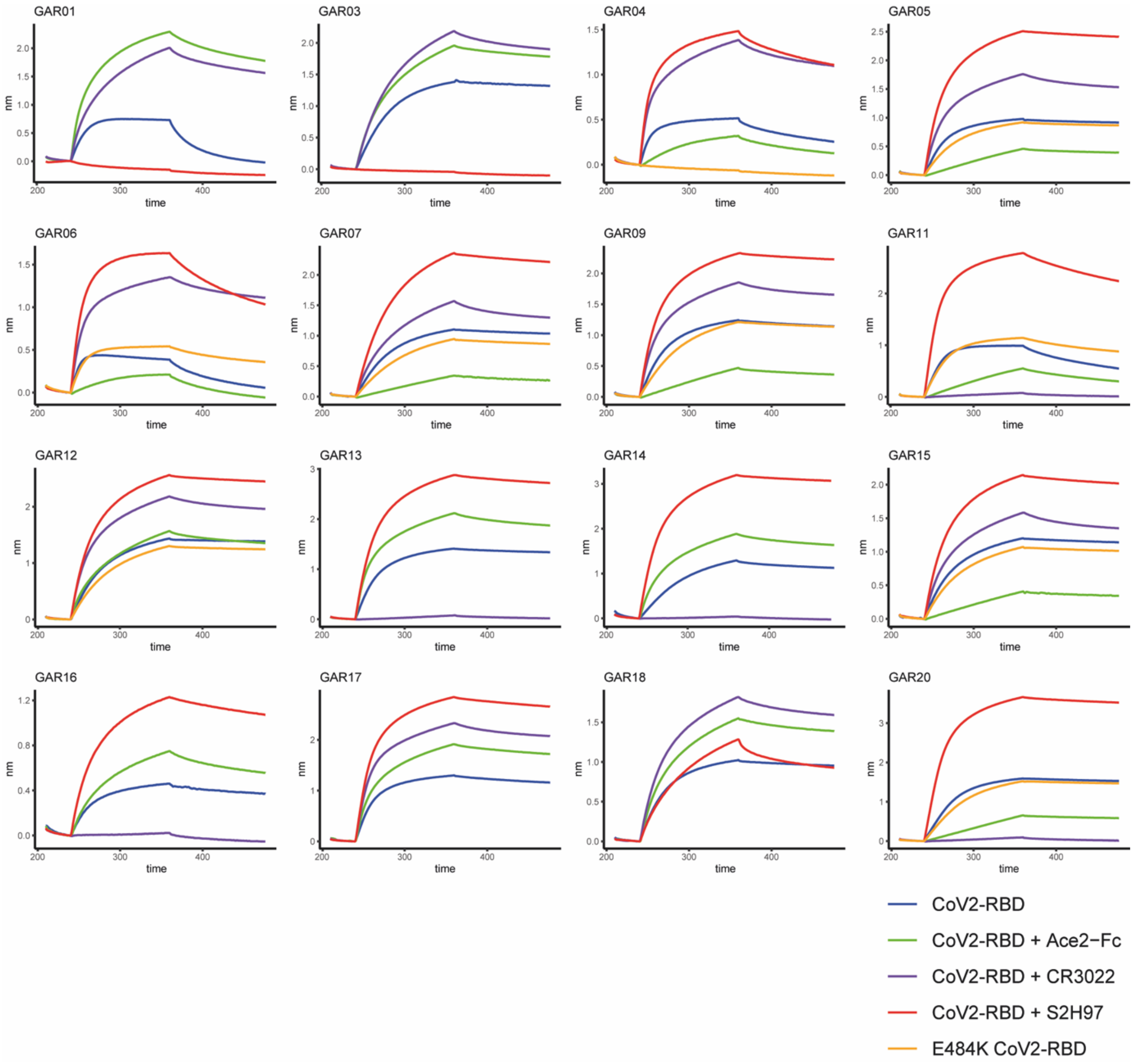

Supplementary Fig. 3: Epitope mapping through BLI competition. Antibodies were initially assessed for binding to the RBD (500 nM) pre-incubated with human ACE2 Fc (1 µM) to identify class 1 and 2 antibodies. Additive binding signal indicates co-binding while a decrease or absence of signal indicates competition. Non-class 1 and 2 antibodies were then evaluated for binding to RBD (500 nM) pre-incubated with CR3022 IgG or with S2H97 IgG (1 µM) to identify the class 4 or 5 antibodies, respectively. Finally, class 1/2 antibodies were evaluated for binding to the RBD E484K mutant (500 nM) to suggest class 2 antibodies.

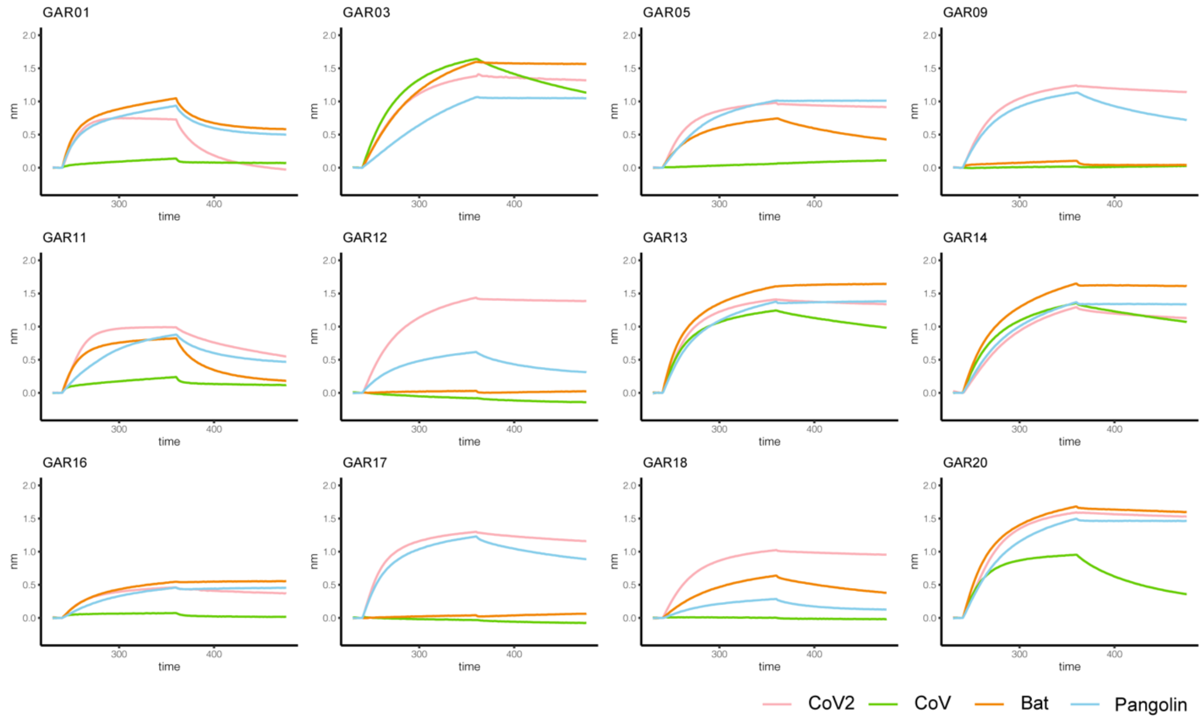

Supplementary Fig. 4: Cross reactivity to other sarbecovirus RBDs. Monoclonal antibodies were evaluated for binding to SARS-CoV-1, Bat RaTG13 and Pangolin CoV by BLI. Biotinylated antibodies were captured onto streptavidin sensors and incubated with the RBDs at 500 nM.

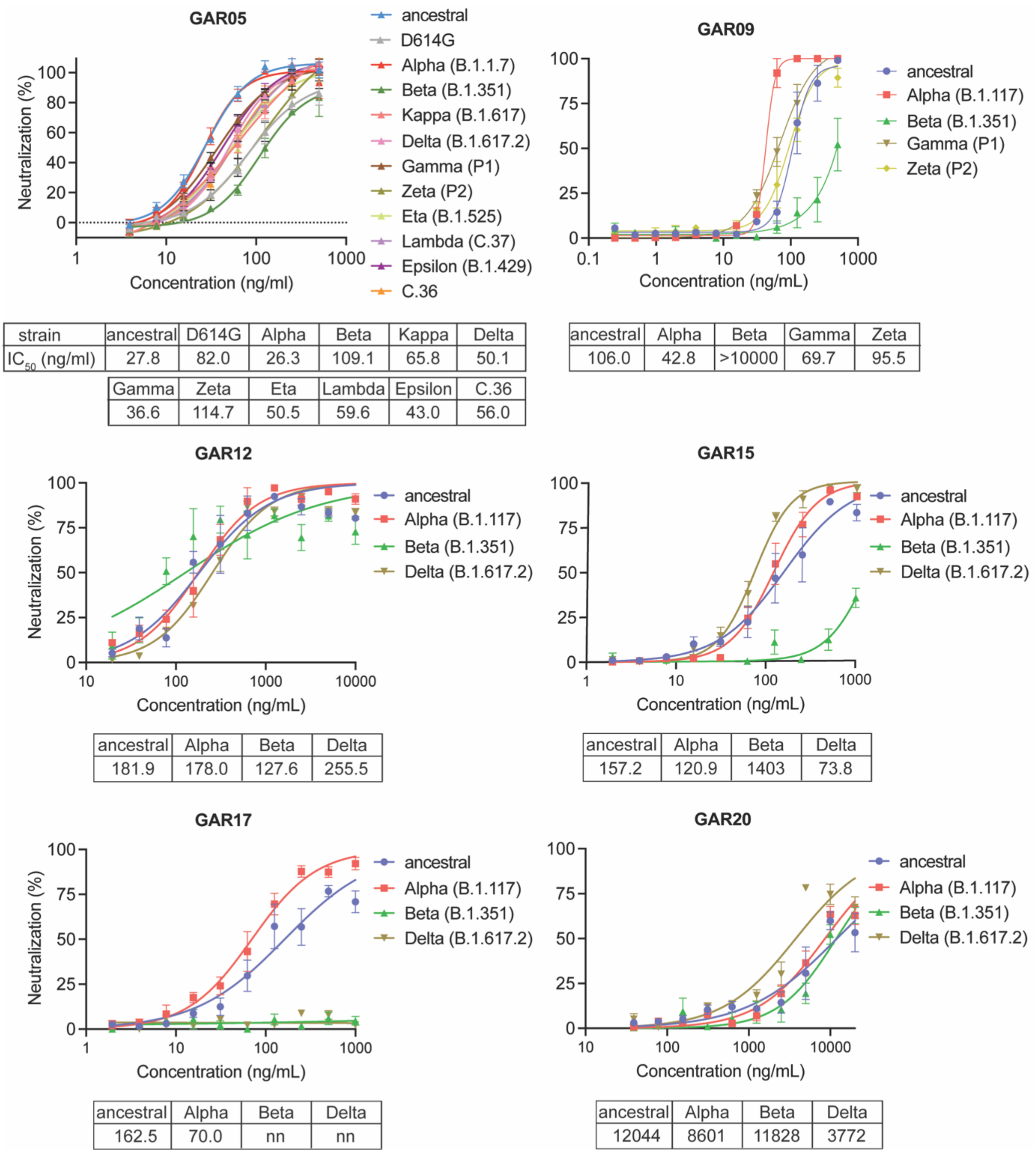

GAR05

strain	ancestral	D614G	Alpha	Beta	Kappa	Delta
IC_{50} (ng/ml)	27.8	82.0	26.3	109.1	65.8	50.1

Gamma	Zeta	Eta	Lambda	Epsilon	C.36
36.6	114.7	50.5	59.6	43.0	56.0

GAR09

ancestral	Alpha	Beta	Gamma	Zeta
106.0	42.8	>10000	69.7	95.5

GAR12

ancestral	Alpha	Beta	Delta
181.9	178.0	127.6	255.5

GAR15

ancestral	Alpha	Beta	Delta
157.2	120.9	1403	73.8

GAR17

ancestral	Alpha	Beta	Delta
162.5	70.0	nn	nn

GAR20

ancestral	Alpha	Beta	Delta
12044	8601	11828	3772

Supplementary Fig. 5: Neutralization of SARS-CoV-2 VOCs. Antibodies were evaluated for *in vitro* live SARS-CoV-2 virus neutralization in Vero E6 cells or HEK293T cells expressing hACE2 (for the Delta variant only). n=4 technical replicates per condition. Data are presented as mean values +/- SD.

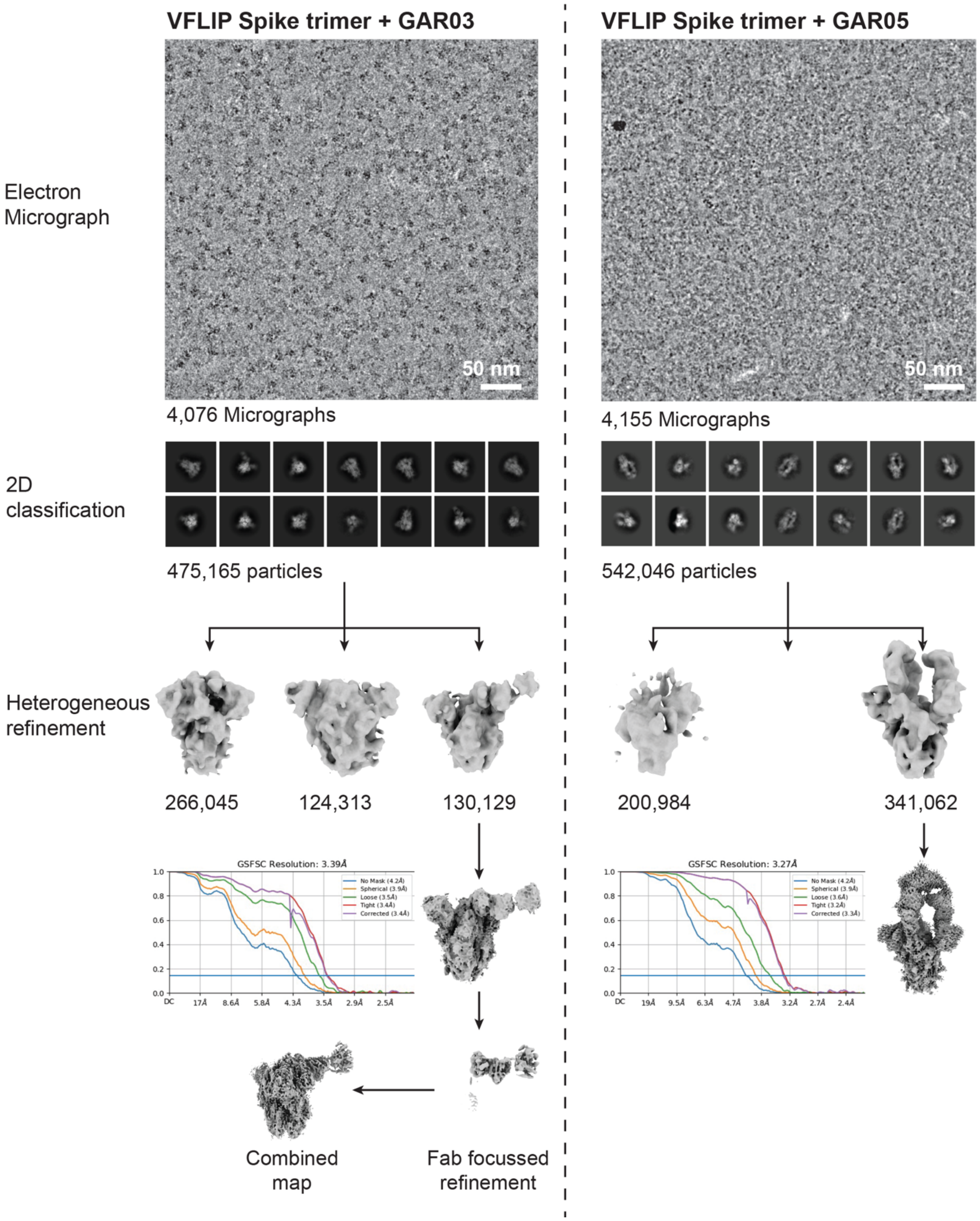

Supplementary Fig. 6: Cryo-EM data processing flowchart. Cryo-EM data processing strategy along with the Fourier shell correction (FSC) curves on the final maps.

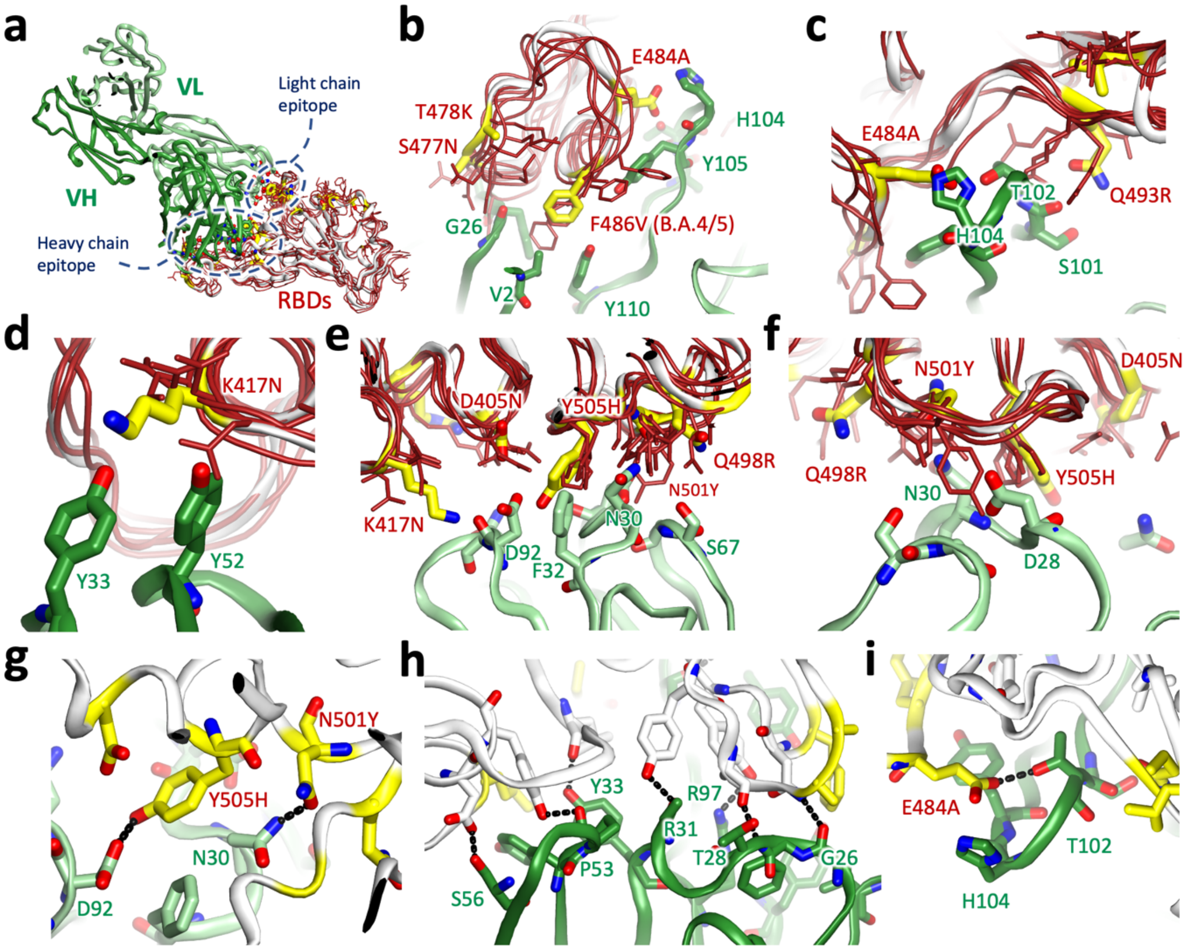

Supplementary Fig. 7: GAR05-RBD superposed with Omicron lineage structures. **a**: GAR05 heavy and light chain (dark and pale green cartoons) bound to ancestral RBD (cream cartoon) superposed with omicron lineage RBDs (thin red cartoon and sticks). Omicron structures include those from lineages B1.1.529, BA.2, and BA.4/5, and come from a variety of conformational contexts*. Residues shown as yellow sticks mark positions mutated in Omicron lineages. The epitopes recognised by GAR05 heavy and light chains are indicated. The perspective is equivalent to that shown in Fig. 2b) Panels **b-d** show close-up views of GAR05 heavy chain residues in proximity to Omicron variant positions. Panel **b** highlights several mutations (S477N, T478K, E484A and F486V (exclusive to B.A.4/5)), which adorn a relatively flexible loop comprising one end of the saddle to which ACE2 binds. Panel **c** focuses on the E484A and Q493R positions, whilst panel **d** highlights the K417N position. Panels **e** and **f** highlight light chain residues in proximity to Omicron mutations. Panels **g-i** detail hydrogen bonds (black dashed lines) between GAR05 and the RBD for the light chain (panel **g**) and the heavy chain (panels **h** and **i**). The bulk of these involve RBD side chains or main-chain residues not mutated in omicron variants (RBD features coloured cream),

with notable exceptions of the light-chain centric Y505H and N501Y (coloured yellow, panel **g**) and heavy-chain centric E484A (panel **i**)

*Omicron structures shown as thin red wire cartoons and sticks comprise; B.1.1.529 in the up and down conformations (PDB 7tgw chains A and B, at 3.0 Å [1]), B.1.1.529 in the exclusively down conformation (PDB 7wp9, chain A, at 2.56 Å [2]), B.1.1.529 in the up conformation and complexed with ACE2 (PDB 7wpa chain A, at 2.77 Å [2]), B.A.2 in the exclusively down conformation (PDB 7ub0, chain A, at 3.31 Å [3]), B.A.4/5 in the exclusively down conformation (PDB 7xnq, chain A, at 3.52 Å [4]), and B.A.4/5 in the up conformation and complexed with ACE2 (PDB 7xwa, chain B, at 3.36 Å [5]). All structures were determined by cryoEM apart from PDB 7xwa, which was determined by X-ray crystallography.

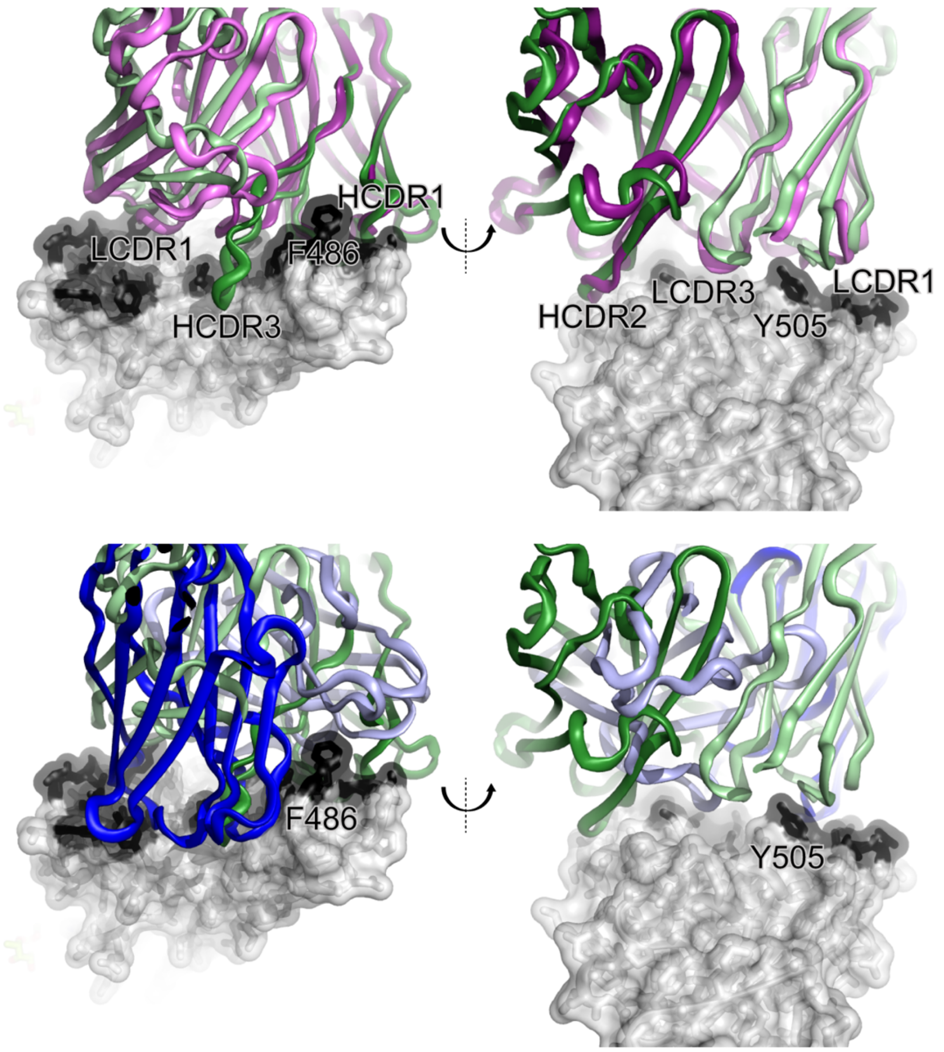

Supplementary Fig. 8: Structural comparison of broadly neutralizing antibodies binding to the class 1/2 epitope (ACE2 mimetics). GAR05 (heavy and light chains as dark and light green cartoon) bound to the RBD (grey transparent surface with ACE2 interface coloured black) superposed with antibodies P2C-1F11 (top panels; dark and light purple for heavy and light chains) and S2K146 (bottom panels; dark and light blue for heavy and light chains). Prominent aromatic residues F486 and Y505 are indicated for reference.

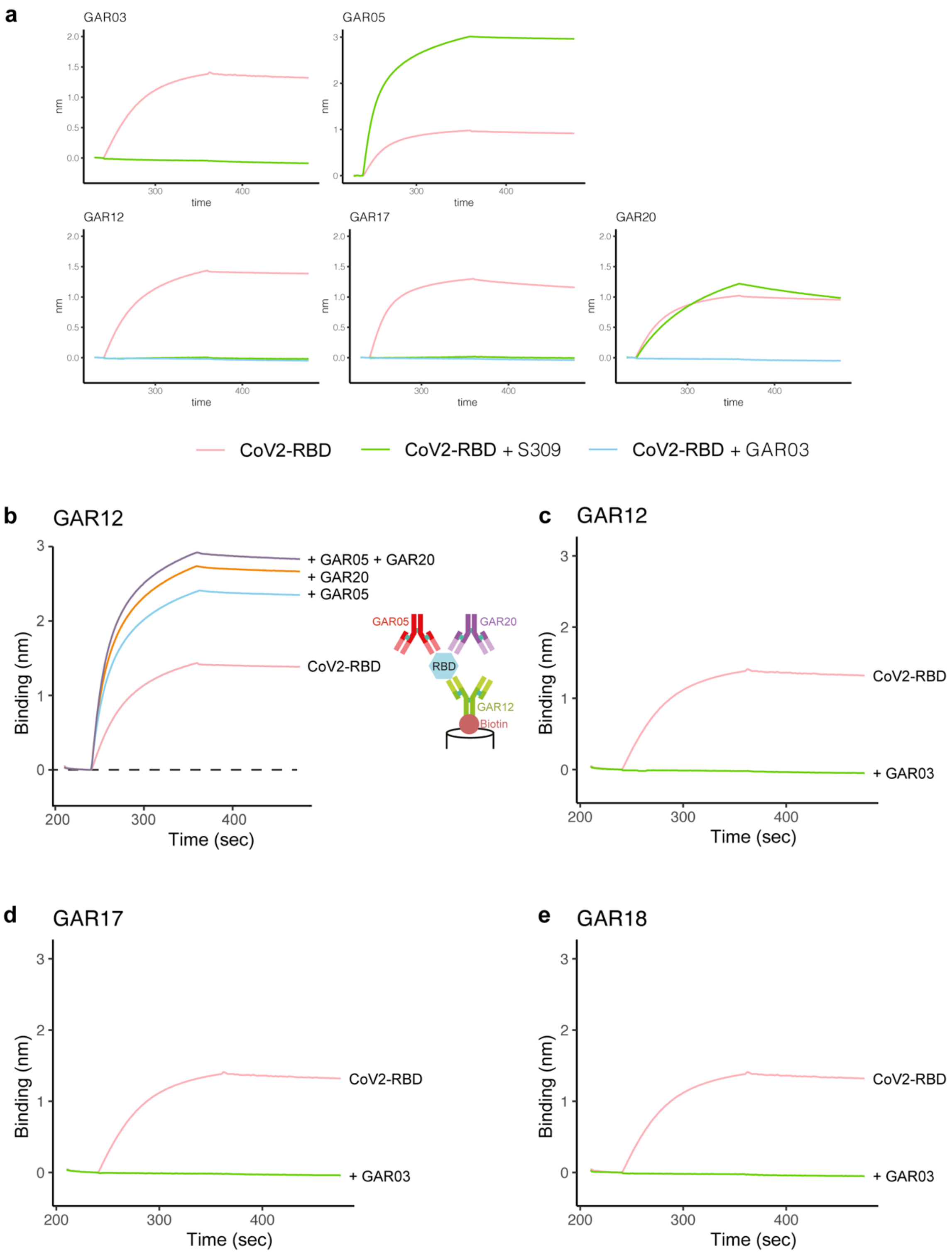

Supplementary Fig. 9: S309 IgG, and GAR-antibody competition. a: To assess antibody epitopes, biotinylated antibodies were captured onto streptavidin sensors and incubated with RBD (500 nM) pre-incubated with S309 IgG (1 µM) or GAR03 IgG (1 µM). **b**: BLI assay demonstrating the non-overlapping binding of GAR05, GAR12 and GAR20 on the surface of the RBD. Biotinylated GAR12 was captured onto streptavidin sensors and incubated with RBD, RBD pre-incubated with GAR05, RBD pre-incubated with GAR20 or RBD pre-incubated with GAR05 and GAR20. Increased binding curves indicate that the

antibodies do not compete. Panels **c-e** demonstrate competitive binding between GAR03 and GAR12 (**c**), GAR17 (**d**) and GAR 18 (**e**).

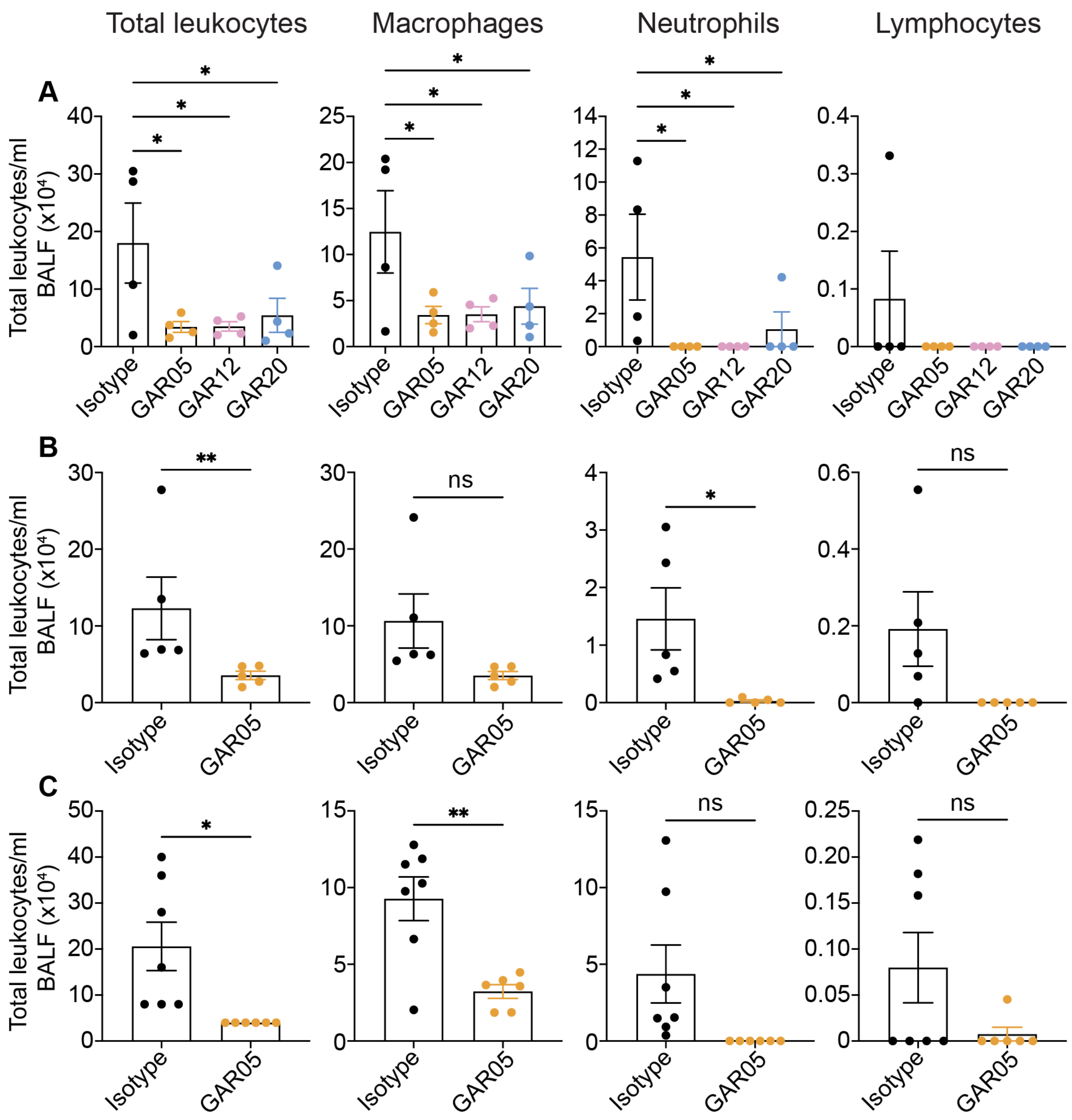

Supplementary Fig. 10: BALF Differential cell counts from *in vivo* studies. Differential leukocytes (macrophages, neutrophils and lymphocytes) cell counts isolated from bronchoalveolar lavage fluid (BALF) of ancestral SARS-CoV-2. (**a**) prophylaxis, and (**b**) therapeutic model, or (**c**) Delta SARS-CoV-2 prophylactic model. (**a**) [n=4 mice per group. One-way ANOVA with an uncorrected Fisher's LSD multiple comparisons test. *p<0.05], (**b**) [n=6 mice per group. Two-tailed unpaired t-test. *p<0.05, **p<0.01], **(c)** [n=7 istoype control mice and n=6 GAR05 treated mice. Two-tailed unpaired t-test. *p<0.05, **p<0.01]. Data are presented as mean values +/- SEM.

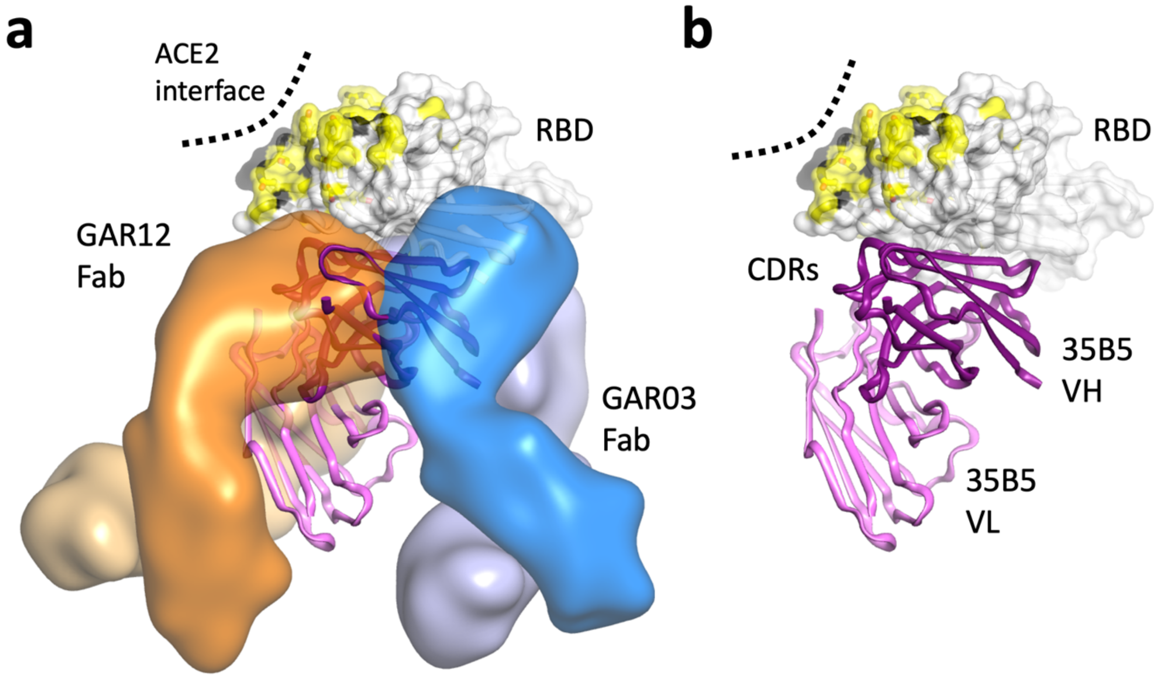

Supplementary Fig. 11: Superposition of GAR03 and GAR12 Fabs with Fv from 35B5. **a**: GAR12 and GAR03 Fabs (heavy and light chains as light/dark orange/blue transparent surfaces) positioned against the RBD (cream molecular surface and cartoon) whereas the ACE2 interface and overlayed omicron mutation positions are shaded black and yellow, respectively. The 35B5 Fv is shown as dark purple and violet cartoons (for heavy and light chains). **b**: The 35B5 antibody binds in an unusual manner with considerable contacts involving heavy-chain framework regions outside of the CDRs.

Supplementary Table 1: Cryo-EM data collection and refinement.

	SARS CoV-2 VFLIP spike trimer + GAR03 (EMDB 25699) (PDB 7T5O)	SARS CoV-2 VLIP spike trimer + GAR05 (EMDB 25700)
**Data collection and processing**		
Magnification	130,000	130,000
Voltage (kV)	300	300
Electron exposure (e^-/Å^2)	50	50
Defocus range (μm)	0.8-1.8	0.8-1.8
Pixel size (Å)	1.08	1.08
Symmetry imposed	C1	C1
Initial particle images (no.)	475,165	542,046
Final particle images (no.)	130,129	341,602
Map resolution (Å)	3.39	3.27
0.143 FSC threshold		
**Refinement**		
Map sharpening *B* factor (Å^2)	-78.3	-88.5
Initial models used (PDB)	6xlu	
Model resolution (Å) 0.5 FSC threshold, Masked/unmasked	7.6/7.8	
B factor (Å^2)		
Protein	199.28	
R.m.s deviations		
Bond lengths (Å)	0.007	
Bond angles (°)	1.384	
Validation		
MolProbity score	1.5	
Clashscore	3.59	
Poor rotamers (%)	0.00	
Ramachandran plot		
Favored (%)	94.95	
Allowed (%)	5.05	
Disallowed (%)	0.00	

Supplementary Table 2: Diffraction data and model refinement statistics. Values in parentheses represent values for the highest resolution shell.

**X-Ray Diffraction Data Collection Statistics**			
Crystal	**GAR05 (CoV-2 RBD + GAR05 Fab)**	**GAR03 (CoV-2 RBD + GAR03 Fab + 10G4 Fab)**	**GAR12 (CoV-2RBD + GAR12 Fab)**
Wavelength	0.9537	0.9537	0.9537
Spacegroup	P 3_2 2 2	P 2_1 2_1 2_1	I 1 2 1
Unit cell dimensions: a, b, c (Å); *α, β, γ*, (°)	111.29, 111.29, 112.86; *90.00, 90.00, 120.00*	55.24, 102.08, 201.82; *90.00, 90.00, 120.00*	93.61, 41.95, 183.03; *90.00, 97.08, 90.00*
Resolution range	3.17-49.91 (3.18-3.40)	2.73-49.48	2.25-48.08 (2.25-2.32)
Total reflections	234263 (10010)	372048 (39714)	221099 (20451)
Unique reflections	13965 (2418)	31192 (3918)	34125 (3150)
Completeness	99.3 (96.5)	99.4 (95.8)	100.0 (100.0)
Multiplicity	16.8 (16.6)	11.9 (10.1)	6.5 (6.5)
Average (I/σ(I))	21.4 (2.9)	12.8 (1.7)	17.0 (1.9)
Mean half set correlation, $CC_{1/2}$	0.998 (0.866)	0.998 (0.672)	0.999 (0.710)
Rmeas (all I+ and I-)	0.079 (1.137)	0.156 (1.384)	0.060 (0.929)
Rpim (all I+ and I-)	0.027 (0.392)	0.047 (0.455)	0.025 (0.394)
Wilson B (Å^2)	126	54.5	48.7
**Refinement and Model Statistics**			
R_{work}/R_{free}	0.237/0.308	0.223/0.269	0.211/0.254
Complexes/asu	1	1	1
Additional Fab	none	Antibody 10G4*	none
Atoms protein	4503	7883	4767
B average protein (Å^2)	134.4	64.13	72.06
B average RBD(Å^2)	134.6	53.51	54.4
B average Heavy (Å^2)	145.3	GAR03: 68.32<br>10G4: 63.41	77.35
B average Light (Å^2)	123.8	GAR03: 74.19<br>10G4: 61.14	84.29
RMSD bond lengths (Å)	0.003	0.003	0.009
RMSD bond angles (°)	0.809	0.706	0.951
Ramachandran Outliers (%)	0.49	0.86	0.33
Ramachandran Favored (%)	92.12	93.50	95.28
**PDB entry**	**7t72**	**8dxu**	**8dxt**

Supplementary Table 3. GAR antibodies described in the study.

Antibody name	VH/VL germlines	VH CDR3 length (Kabat numbering)	Germline mutations
**GAR01**	IGHV3-30 & IGKV1-33	13	6 in VH, 1 in VL
**GAR03**	IGHV1-8 & IGLV3-21	12	4 in VH, 4 in VL
**GAR04**	IGHV1-2 & IGKV4-1	23	2 in VH, 1 in VL
**GAR05**	IGHV3-66 & IGKV1-33	14	8 in VH, 3 in VL
**GAR06**	IGHV2-70 & IGLV2-11	12	2 in VH, 4 in VL
**GAR07**	IGHV3-66 & IGKV1-33	9	4 in VH, 1 in VL
**GAR09**	IGHV3-66 & IGKV1-33	8	3 in VH, 2 in VL
**GAR11**	IGHV1-69 & IGLV2-14	17	10 in VH, 2 in VL
**GAR12**	IGHV3-23 & IGKV1-5	15	6 in VH, 1 in VL
**GAR13**	IGHV3-13 & IGKV1-39	15	4 in VH, 1 in VL
**GAR14**	IGHV3-13 & IGKV1-39	17	5 in VH, 4 in VL
**GAR15**	IGHV3-53 & IGKV1-9	10	3 in VH, 4 in VL
**GAR16**	IGHV3-13 & IGKV1-39	21	4 in VH, 4 in VL
**GAR17**	IGHV3-23 & IGLV1-47	17	6 in VH, 5 in VL
**GAR18**	IGHV4-59 & IGKV3-11	15	4 in VH, 2 in VL
**GAR20**	IGHV3-34 & IGLV3-21	12	6 in VH, 3 in VL

Supplementary Table 4. GAR Antibody Sequences

**GAR01**	IGHV3-30 & IGKV1-33
VH	QVQLVQSEGGVVQPGRSLRLSCTASGFTFSSYGMHWVRQAPGKGLEWVALISYDGSDKYYADSVKGRFTISRDNSKNTLYLQMNSLRPEDTAVFYCAKGGWYGSSSGFDYWGQGTLVTVSS
VL	DIQMTQSPSSLSASVGDRVTITCQASQDISNYLNWYQQKPGKAPKLLIYDASNLETGVPSRFSGSGSGTDFIFTISSLQPEDIATYYCQQYHDVPRTFGQGTKVEIK
**GAR03**	IGHV1-8 & IGLV3-21
VH	QVQLVQSGAEVKKPGASVKVSCKASGYTFSSYDINWVRQAPGQGLEWMGWMSPNSGNTGYAQKFQGRVTMTRSTSMSTAYMELSSLRSEDTAVYFCARMSSSLTNYLDYWGQGTLVTVSS
VL	SYVLTQPPSVAVAPGQTARIRCGENDIGSKNVHWYQQKSGQAPVLVVYDDSDRPSGIPERFSGSNSGNTATLTISRVEAGDEADYYCQVWDSTGDHPDVVFGGGTKLTVL
**GAR04**	IGHV1-2 & IGKV4-1
VH	QVQLVQSGAEVKKSGASVKVSCKASGYTFTGYHMHWVRQAPGQGLEWMGWINPKSDGTNSAQKFQGRVTMTRDTSISTAYMELSRLRSDDTAVYYCARLAVREYYDNSGFYRKTTDAFDLWGQGTMVTVSS
VL	DIVMTQSPDSLAVSLGERATINCKSSQSVLYNSNNKNYLAWYQQKPGQPPKLLIYWASTRESGVPDRFSGSGSGTDFTLTINSLQAEDVAVYYCQQYSITTPPITFGQGTRLEIKR
**GAR05**	IGHV3-66 & IGKV1-33
VH	EVQLVQSGGDLVQPGGSLRLSCAVSGFTVSRNYMTWVRQAPGRGLEWVSLIYPGGSAFYADSVKGRFTISRDNSKNTLYLQMNSLRVEDTAVYYCARDPVSTGHYHDSDYWGQGTLVTVSS
VL	DIQMTQSPSALSASVGDRVTITCQASQDINKFLNWYQQKPGKAPKLLIYDASNLETGVPSRFSGGGSGTDFTFTISSLQPEDIATYYCHQYDNLPRTFGQGTKVELK
**GAR06**	IGHV2-70 & IGLV2-11
VH	QVTLRESGPALVKPTQTLTLTCTFSGFSVSSAGMCVSWIRQPPGKALEWLARIDWDDDKYYSTSLRTRLTISTDTSKNQVVLTMTNMDPVDTATYYCARTTMTTSYYFDSWGQGTLVTVSS

VL	QSALTQPRSVSGSPGQSVTISCTGTSSDVGGYDYVSWYQHHPGKAPKVV<br>LYDVTKRPSGVPDRFSGSKSGNTASLTISGLQAEDEADYYCCSYAGSYA<br>GVFGTGTKVTVLG
**GAR07**	IGHV3-66 & IGKV1-33
VH	EVQLVQSGGGLVQPGGSLRLSCAASEFIVSSNYMSWVRQAPGKGLEWV<br>SVIYSGGSTFYADSVKGRFTISRDNSKNTLHLQMNSLRVEDTAVYYCAR<br>DRGDRFDYWGQGTLVTVSS
VL	DIVMTQSPSSLSASVGDRVTITCQASQGISNYLNWYQQKPGKAPKLLIYG<br>ASNLETGVPSRFSGSGSGTDFTFTISSLQPEDIATYYCQQYDNLPSFTFGP<br>GTKVDIK
**GAR09**	IGHV3-66 & IGKV1-33
VH	EVQLVESGGGLVQPGGSLRLSCAASGFTVSYNYMSWVRQAPGKGLEW<br>VSLIYSGGSTYYADSVKGRFTISRDSSKNTLYLQMNSLRVEDTAVYYCA<br>REVPSRSGWSGGQGTLVTVSS
VL	DIVMTQSPSSLSASVGDRVTITCQASQDMNKYLNWYQQKPGKAPKLLIY<br>DASNLETGVPSRFSGSGSGTNFTFTISSLQPEDIATYYCQQYDNLPPVFGP<br>GTKVEIK
**GAR11**	IGHV1-69 & IGLV2-14
VH	EVQLVQSGTEVKKPGSSVKVSCEASGGTFSKYAITWVRQAPGQGLEWM<br>GGIIPIFGTANYAQKFQDRVKITADEITSIAYMELNSLSSEDTAVYYCARD<br>NRGRGFGEILSYYYGMDVWGQGTTVTVSS
VL	SALTQPASVSGSPGQSITISCTGTSSDVGGYNYVSWYQQHPGKAPKLMIY<br>DVATRPSGVSDRFSGSKSGNTASLTISGLQAEDEADYYCSSYTSSNTLAF<br>GGGTKLTVLGQ
**GAR12**	IGHV3-23 & IGKV1-5
VH	EVQLVQSGGGLVQPGGSLRLSCAASGFTFSSYAMSWVRQAPGKGLEWV<br>LSISGSGGSTYYADSVKGRFTISIDNSKKTVYLQMNSLRAEDTAVYYCAK<br>SGEQQLVQAEYFHHWGQGTLVTVSS
VL	DIMTQSPSTLSASVGDRVTITCRASQSIGRWLAWYQQKPGKAPKLLIYDA<br>SSLESGVPSRFSGSGFGTEFTLTISSLQPDDFATYYCQQCDSLLTFGGGTK<br>LEIK
**GAR13**	IGHV3-13 & IGKV1-39
VH	EVQLVESGGGLVQPGGSLRLSCAASGFTFSNYDMHWVRQVTGKGLEW<br>VSLIGTSGDTYYPGSVKGRFTISRDNAKNSLYLQMNSLRADDTAVYYCV<br>RAAFDDHNIYSWFDSWGQGTLVTVSS

VL	DIQMTQSPSSLSASVGDRVTITCRASQSIGRYLNWYQQKPGKAPKLLIYTASSLQSGVPSRFSGSGSGTEFTLTISSLQPEDFATYYCQQSYNIPPLTFGGGTKVEIK
**GAR14**	IGHV3-13 & IGKV1-39
VH	EVQLVESGGGLVQPGGSLRLSCAASGFTFSRYDMHWVRQTTGKGLEWVSAIGTAGDTYYPDSVKGRFTISRENAKNSLHLQMNSLRVGDTAVYFCARAVDCSDISCYSNWYFDLWGRGTLVTVSS
VL	DIQLTQSPSSLSASVGDRVTITCRASQTISSFLNWYQQKPGKAPHLLIYAASSLQSGVPSRFSGSGSGTDFTLTITNLQPEDFATYYCQQSYSTPPITFGQGTRLEIK
**GAR15**	IGHV3-53 & IGKV1-9
VH	EVQLVESGGGLVQPGGSLRLSCAASGLTVSSNYMSWVRQAPGKGLEWVSVIYSGGSTFYADSVKGRFTISRHNSKNTLYLQMNSLRGDDTAVYYCARDLIYYGMDVWGQGTTVTVSS
VL	DIVMTQSPSSLSASVGDRVTITCRASQGISSSLAWYQQNPGKPPKLLIYAASTLQSGVPSRFSGSGSGTDFTLTISSLQPEDFATYYCQQLNSDLYTFGQGTKVEIK
**GAR16**	IGHV3-13 & IGKV1-39
VH	EVQLVESGGGVVQPGGSLRLSCAASGFTFSNYDMHWVRQATGKGLEWVSAIGTAGDTYYPDSVKGRFTISRDNAKNSLYLQMNSLRAEDTAVYYCTRVVHSSDWTSWSAGIHNWFDSWGQGTLVTVSS
VL	DIVMTQSPSSLSASVGDRVSITCRTSQSIRTYLNWYQQKPGKAPKLLIYASSSLQSGVPSRFSGSGSGTDFTLSISSLQPEDFATYYCQQSYSMPPWTFGQGTKLEIK
**GAR17**	IGHV3-23 & IGLV1-47
VH	EVQLVESGGGLGQPGGSLRLSCAASGFNFSNYAMSWVRQAPGKGLEWISTVGGSGDSTYYADSVKGRFTISIDNSKNRLYLQMNSLRSEDTAVYYCAKELAVMPPYFYYYAMDVWGQGTTVTVSS
VL	QPVLTQPPSASGTPGQRVTISCSGTTSNVGSTYVYWYQQLPGTAPKLLIYRYNQRPSGVPDRFSGSKSGTSASLVISGLRSDDEAVYYCATWDDSRHVVFGGGTKLTVL
**GAR18**	IGHV4-59 & IGKV3-11
VH	QVQLQESGPGLVKPSETLSLTCTVSGGSISDYYWSWIRQPPGKGLEWIGYIHGSGGTNYNPSLKSRVTILVDTSKNELSLKLSSVTTADTAVYYCARDDMISGLGYYGMDVWGQGTTVTVSS

VL	EIVMTQSPATLSLSPGERVTLSCRASQSVSSYLAWYQQKPGQAPRLLIYD ASNRATGIPARFSGSGSGTDFTLTISSLEPEDFAVYYCQQRSNWPSTFGPG TKVDIK
**GAR20**	IGHV3-34 & IGLV3-21
VH	QVQLQQWGAGLLKPSETLSLTCAVYGGSFSDFYWSWIRQPPGKGLEWI GQIDHSGSPNYNPSLKSRVTMSVDTSKNHFSLRLTSMTAADTAVYYCAK GPRYGSGSFDPLGQGTLVTVSS
VL	SYELTQPPSVSVAPGQTARITCGGDNIGSKTVHWYQQKPGQAPVLVVYD DGDRPSGIPERFSGSNPGNTATLTISRVEAGDEADYYCQVWDSSSDSVVF GGGTELTVL

1 Ye, G., Liu, B. & Li, F. Cryo-EM structure of a SARS-CoV-2 omicron spike protein ectodomain. *Nature Communications* **13**, 1214 (2022). https://doi.org:10.1038/s41467-022-28882-9

2 Yin, W. *et al.* Structures of the Omicron spike trimer with ACE2 and an anti-Omicron antibody. *Science* **375**, 1048-1053 (2022). https://doi.org:doi:10.1126/science.abn8863

3 Stalls, V. *et al.* Cryo-EM structures of SARS-CoV-2 Omicron BA.2 spike. *Cell Reports* **39**, 111009 (2022). https://doi.org:https://doi.org/10.1016/j.celrep.2022.111009

4 Cao, Y. *et al.* BA.2.12.1, BA.4 and BA.5 escape antibodies elicited by Omicron infection. *Nature* **608**, 593-602 (2022). https://doi.org:10.1038/s41586-022-04980-y

5 Kimura, I. *et al.* Virological characteristics of the SARS-CoV-2 Omicron BA.2 subvariants, including BA.4 and BA.5. *Cell* **185**, 3992-4007.e3916 (2022). https://doi.org:https://doi.org/10.1016/j.cell.2022.09.018
