# Supplementary material for: Broadly neutralizing SARS-CoV-2 antibodies through epitope-based selection from convalescent patients
Source: Nat Commun. 2023 Feb 8;14:687. doi: 10.1038/s41467-023-36295-5 (PMC9907207; doi:10.1038/s41467-023-36295-5)
Supplement: Supplementary file 2 — Reporting Summary [file 41467_2023_36295_MOESM2_ESM.pdf]

nature portfolio

Corresponding author(s): *Double-blind peer review submissions: write DBPR and your manuscript number here instead of author names.*

Last updated by author(s): *YYYY-MM-DD*

Reporting Summary

Nature Portfolio wishes to improve the reproducibility of the work that we publish. This form provides structure for consistency and transparency in reporting. For further information on Nature Portfolio policies, see our Editorial Policies and the Editorial Policy Checklist.

Statistics

For all statistical analyses, confirm that the following items are present in the figure legend, table legend, main text, or Methods section.

n/a	Confirmed	
☐	☒	The exact sample size (*n*) for each experimental group/condition, given as a discrete number and unit of measurement
☐	☒	A statement on whether measurements were taken from distinct samples or whether the same sample was measured repeatedly
☐	☒	The statistical test(s) used AND whether they are one- or two-sided<br>*Only common tests should be described solely by name; describe more complex techniques in the Methods section.*
☐	☒	A description of all covariates tested
☒	☐	A description of any assumptions or corrections, such as tests of normality and adjustment for multiple comparisons
☐	☒	A full description of the statistical parameters including central tendency (e.g. means) or other basic estimates (e.g. regression coefficient) AND variation (e.g. standard deviation) or associated estimates of uncertainty (e.g. confidence intervals)
☐	☒	For null hypothesis testing, the test statistic (e.g. *F*, *t*, *r*) with confidence intervals, effect sizes, degrees of freedom and *P* value noted<br>*Give P values as exact values whenever suitable.*
☒	☐	For Bayesian analysis, information on the choice of priors and Markov chain Monte Carlo settings
☒	☐	For hierarchical and complex designs, identification of the appropriate level for tests and full reporting of outcomes
☒	☐	Estimates of effect sizes (e.g. Cohen's *d*, Pearson's *r*), indicating how they were calculated

Our web collection on statistics for biologists contains articles on many of the points above.

Software and code

Policy information about availability of computer code

Data collection	FACS Diva v6.1.3 (Becton Dickinson)<br>XDS (version 0.6.5.2)<br>Blitz Pro v1.2.1.3 (Forte Bio)
Data analysis	FlowJo v10.7.1 (Becton Dickinson)<br>Pointless (version 1.12.12)<br>Aimless (version 0.7.7)<br>Phaser (version 2.8.3)<br>Coot (version 0.9.6)<br>Refmac (version 5.8.0267)<br>Phenix-refine (version 1.11.1-2775)<br>Prism v9.4.0 (Graphpad Software)<br>Snapgene v6.1.1 (GSL Biotech LLC)

For manuscripts utilizing custom algorithms or software that are central to the research but not yet described in published literature, software must be made available to editors and reviewers. We strongly encourage code deposition in a community repository (e.g. GitHub). See the Nature Portfolio guidelines for submitting code & software for further information.

Data

Policy information about availability of data

All manuscripts must include a data availability statement. This statement should provide the following information, where applicable:

- Accession codes, unique identifiers, or web links for publicly available datasets
- A description of any restrictions on data availability
- For clinical datasets or third party data, please ensure that the statement adheres to our policy

Crystallography
The crystal structures described in the manuscript are available for download from the Protein Data Bank, accession codes 7t72, 8dxu and 8dxt. Structures for these entries were solved and refined in essentially the same manner, as follows. Diffraction data were indexed and integrated using XDSME (version 0.6.5.2). Scaling and merging were performed with Pointless (version 1.12.12) and Aimless (version 0.7.7). Molecular replacement was performed using Phaser (version 2.8.3). Maps were inspected and real space refinement performed using Coot (version 0.9.6). Refinement was performed using Refmac (version 5.8.0267) and Phenix-refine (version 1.11.1-2775). Figures were prepared using Pymol (version 1.20).

Human research participants

Policy information about studies involving human research participants and Sex and Gender in Research.

Reporting on sex and gender	Data not collected for this study
Population characteristics	see above
Recruitment	Patient PBMC samples were accessed via the COSIN Study (New South Wales COVID-19 patient cohort). ClinicalTrials.gov ID: NCT04383652
Ethics oversight	The protocol was approved by the Human Research Ethics Committees of the Northern Sydney Local Health District and the University of New South Wales, NSW Australia (ETH00520) and was conducted according to the Declaration of Helsinki and International Conference on Harmonization Good Clinical Practice (ICH/GCP) guidelines and local regulatory requirements. Written informed consent was obtained from all participants before study procedures.

Note that full information on the approval of the study protocol must also be provided in the manuscript.

Field-specific reporting

Please select the one below that is the best fit for your research. If you are not sure, read the appropriate sections before making your selection.

☒ Life sciences ☐ Behavioural & social sciences ☐ Ecological, evolutionary & environmental sciences

For a reference copy of the document with all sections, see nature.com/documents/nr-reporting-summary-flat.pdf

Life sciences study design

All studies must disclose on these points even when the disclosure is negative.

Sample size	From a priori one way ANOVA (fixed effects) power analysis of previous data sets, we have calculated that due to the variability of in vivo systems, to achieve 20-30% differences in groups with a confidence of 95% experimental groups will require minimum 4 mice per group.
Data exclusions	No data excluded
Replication	Two variant strains of SARS-CoV2 were utlised in 3 exerimental models with 16 isotype control and 16 GAR05 treated mice across the 3 models over the course of approximately 1 year with comparable data generate in each instance.
Randomization	Cohorts of mice were randomly assigned to each experimental condition
Blinding	Blinded group allocation was not possible as all therapeutic interventions were performed by a single operator.

Reporting for specific materials, systems and methods

We require information from authors about some types of materials, experimental systems and methods used in many studies. Here, indicate whether each material, system or method listed is relevant to your study. If you are not sure if a list item applies to your research, read the appropriate section before selecting a response.

Materials & experimental systems

n/a	Involved in the study
☐	☒ Antibodies
☐	☒ Eukaryotic cell lines
☒	☐ Palaeontology and archaeology
☐	☒ Animals and other organisms
☒	☐ Clinical data
☒	☐ Dual use research of concern

Methods

n/a	Involved in the study
☒	☐ ChIP-seq
☐	☒ Flow cytometry
☒	☐ MRI-based neuroimaging

Antibodies

Antibodies used

The following primary antibodies were sourced from Becton Dickinson (BD): anti-human CD21 BV421 (clone B-ly4, Cat: 562966), anti-human IgD BV510 (clone IA6-2, Cat: 563034), anti-human CD19 BV711 (clone SJ25C1, Cat: 563036), anti-human CD20 APC-H7 (clone L27, Cat: 641396), anti-human IgG BV786 (clone G18-145, Cat: 564230), anti-human CD27 PE-CF594 (clone M-T271, Cat: 562297), anti-human CD38 PE-Cy7 (clone HIT2, Cat: 560677), anti-human CD3 BB700 (clone OKT3, Cat: 566818).

The following HRP-conjugated secondary antibody was sourced from Jackson Immuno Research : Peroxidase Affinity Pure Goat AntiHumanIgG(H+L) (Cat No. 109-035-088).

Validation

anti-human CD21 BV421, clone B-ly4, 562966, BD was validated by the supplier via flow cytometry
anti-human IgD BV510, clone IA6-2, 563034, BD was validated by the supplier via flow cytometry
anti-human CD19 BV711, clone SJ25C1, 563036, BD was validated by the supplier via flow cytometry
anti-human CD20 APC-H7, clone L27, 641396, BD was validated by the supplier via flow cytometry
anti-human IgG BV786, clone G18-145, 564230, BD was validated by the supplier via flow cytometry
anti-human CD27 PE-CF594, clone M-T271, 562297, BD was validated by the supplier via flow cytometry
anti-human CD38 PE-Cy7, clone HIT2, 560677, BD was validated by the supplier via flow cytometry
anti-human CD3 BB700, clone OKT3, 566818, BD was validated by the supplier via flow cytometry
Peroxidase Affinity Pure Goat Anti-Human IgG (H+L) (Cat No. 109-035-088) validated by the supplier by western blot and ELISA

Eukaryotic cell lines

Policy information about cell lines and Sex and Gender in Research

Cell line source(s)

Expi293, ExpiCHO and HEK293T cells lines were sourced from Thermo Scientific.
VeroE6 cells (CRL-1586) were sourced from ATCC

Authentication

Cell lines sourced from Thermo Scientific were issued with appropriate certificates of analysis.

The Garvan Molecular Genetics facility at the Garvan Institute of Medical Research performed VeroE6 cell line authentication on all human cell lines used. DNA from each cell line was analysed for short tandem repeat loci using the PowerPlexR 18D System. Tested cells were >80% identical, indicating they originated from the cell line specified

Mycoplasma contamination

All cell lines tested negative for mycoplasma contamination

Commonly misidentified lines
(See ICLAC register)

No misidentified cell lines were used in this study.

Animals and other research organisms

Policy information about studies involving animals; ARRIVE guidelines recommended for reporting animal research, and Sex and Gender in Research

Laboratory animals

6-8 week old K18-hACE2 mice (B6.Cg-Tg(K18-hACE2)2Prlmn/J, stock Nb. 034860, Jackson Lab) were housed at 21C, 52% humidity on a 12-hour dark/light cycle (dark 7pm-7am)

Wild animals

none used

Reporting on sex

female mice only used

Field-collected samples

no field collected samples used

Ethics oversight

Sydney Local Health District (SLHD) Animal Ethics and Welfare Committee

Note that full information on the approval of the study protocol must also be provided in the manuscript.

Flow Cytometry

Plots

Confirm that:

- [x] The axis labels state the marker and fluorochrome used (e.g. CD4-FITC).
- [x] The axis scales are clearly visible. Include numbers along axes only for bottom left plot of group (a 'group' is an analysis of identical markers).
- [x] All plots are contour plots with outliers or pseudocolor plots.
- [x] A numerical value for number of cells or percentage (with statistics) is provided.

Methodology

Sample preparation	Cryopreserved PBMCs from five convalescent patients were thawed and suspended in pre-warmed RPMI-1640 media containing 10% FBS (sigma), 2 mM L-glutamine, 50 IU/ml penicillin and 50 µg/ml streptomycin. A maximum of 1x10^7 cells were resuspended in Fixable Viability Stain 700 (BD) diluted to 1:1000 in PBS and incubated at 4°C for 20 min. Cells were washed twice with FACS buffer (PBS + 0.1% BSA) and incubated with Human Fc block (5µl per 2x10^6 cells; BD) at room temperature for 10 min. Cells were then washed twice with FACS buffer and resuspended with tetramers at 1µg/ml (per tetramer) and incubated at 4°C for 30 min and washed twice more with FACS buffer. Cells were finally suspended in a cell surface staining mix containing (per test): 50 µl brilliant staining buffer (BD), and all antibodies listed above. Surface staining incubation was performed at 4°C for 30 min, washed twice and resuspended in FACS buffer for sorting.
Instrument	FACS Aria III, Becton Dickinson (BD)
Software	FACS Diva v6.1.3 (BD), FlowJo v10.7.1 (BD)
Cell population abundance	Relative abundance of relevant cells within the sorted fraction was 100% as B cells were index sorted. Purity of single cells selected for antibody cloning was determined by single-cell BCR sequencing.
Gating strategy	General Gating: SSC-A/FSC-A [Lymphocyte Gate]-> FSC-H/FSC-A [Singlet gate]-> BD Fixable Viability Stain 700/FSC-A [Viable cell population]-> CD3 BB700 / CD19 BV711 [CD3- cells]-> CD20 APC-H7 / CD19 BV711 [CD19+CD20+ B cells]-> IgG BV786 / CD10 BV605 [CD10- B cells]-> CD27 PE-CF594 / IgD BV510 [IgD- B cell]<br><br>Sort 1: [IgD- B cell as above]-> WT CoV2 RBD tetramer PE / WT CoV2 SPIKE tetramer APC [index sort PE+ OR APC+ cells]<br>Sort 2: [IgD- B cell as above]-> WT RBD tetramer PE / CoV1 RBD tetramer BB515 [index sort PE+ OR BB515+ cells]<br>Sort 3: [IgD- B cell as above]-> WT RBD tetramer PE / IgG BV786 [index sort PE+ cells]<br><br>Boundaries between "positive" and "negative" staining as defined by observation of bi-modal stained populations and back-gating B cell specific surface markers to CD3+ cells

- [x] Tick this box to confirm that a figure exemplifying the gating strategy is provided in the Supplementary Information.
